# Nominal Operating Envelope of Pod and Pen Style Electronic Cigarettes

**DOI:** 10.3389/fpubh.2021.705099

**Published:** 2021-08-17

**Authors:** Edward C. Hensel, Nathan C. Eddingsaas, Qutaiba M. Saleh, Shehan Jayasekera, S. Emma Sarles, Mahagani Thomas, Bryan T. Myers, Gary DiFrancesco, Risa J. Robinson

**Affiliations:** ^1^Department of Mechanical Engineering, Rochester Institute of Technology, Rochester, NY, United States; ^2^Department of Chemistry and Materials Science, Rochester Institute of Technology, Rochester, NY, United States; ^3^Department of Electrical and Computer Engineering, Rochester Institute of Technology, Rochester, NY, United States; ^4^Department of Mechanical and Industrial Engineering, Rochester Institute of Technology, Rochester, NY, United States; ^5^Department of Biomedical and Chemical Engineering, Rochester Institute of Technology, Rochester, NY, United States

**Keywords:** operating envelope, E-cigarette, electronic nicotine delivery system, pod-style, pen-style

## Abstract

Many Electronic Nicotine Delivery Systems (ENDS) employ integrated sensors to detect user puffing behavior and activate the heating coil to initiate aerosol generation. The minimum puff flow rate and duration at which the ENDS device begins to generate aerosol are important parameters in quantifying the viable operating envelope of the device and are essential to formulating a design of experiments for comprehensive emissions characterization. An accurate and unbiased method for quantifying the flow condition operating envelope of ENDS is needed to quantify product characteristics across research laboratories. This study reports an accurate, unbiased method for measuring the minimum and maximum aerosolization puff flow rate and duration of seven pod-style, four pen-style and two disposable ENDS. The minimum aerosolization flow rate ranged from 2.5 to 23 (mL/s) and the minimum aerosolization duration ranged from 0.5 to 1.0 (s) across the ENDS studied. The maximum aerosolization flow rate was defined to be when the onset of liquid aspiration was evident, at flow rates ranging from 50 to 88 (mL/s). Results are presented which provide preliminary estimates for the effective maximum aerosolization flow rate and duration envelope of each ENDS. The variation in operating envelope observed between ENDS products of differing design by various manufacturers has implications for development of standardized emissions testing protocols and data reporting required for regulatory approval of new products.

## Introduction

There is little consistency in puffing regimes being used for ENDS emission studies; studies have used 15 ml/s, 4 s puffs (1), 27 ml/s, 3 s puffs (49), 39 ml/s, 1.8 s puffs (2), 27.5 ml/s, 2 s puffs (3, 4), 17.5 ml/s 2 s puffs (5–7), 10 ml/s, 4 s puffs (8), and in some articles the puffing protocol is unclear (9, 10). It remains unclear how the puffing regimes used relate to the normal range of the device permitted by the manufacturer, or how the puffing regimes correlated with user behavior. Prior work shows that emissions are a strong function of puff flow rate (11), and that puff flow rate and other topography behavior varies widely with individual users and devices (12, 13). To date, no standard emissions outcome measures have been agreed upon, while a wide variety of metrics have been reported. Emissions have frequently been reported as the total condensed aerosol, commonly referred to as the Total Particulate Matter (TPM) yield per puff [Y_TPM_, (mg/puff)] and the Harmful and Potentially Harmful Constituents (HPHC) Yield [Y_HPHC_, (mg/puff), i.e., the mass of selected HPHCs per number of puffs] (2, 3, 14–32). Some studies have reported emissions in terms of the HPHC mass ratio, f_HPHC_ (mg/mg) (i.e., the mass of selected HPHCs per unit mass of TPM) (17, 26, 30, 31, 33). Previously proposed smoking machine standards such as (34) provided a basis for product comparisons but did not reflect the range of user behaviors observed (35), thus limiting their utility for public health assessments. Similarly, recent vaping machine standards (36) provide some basis for product comparisons, but also do not reflect the range of use behavior anticipated in the natural environment. Yield terms, such as Y_TPM_, are normalized “per puff,” while mass concentration terms, such as C_TPM_, are normalized by the puff volume expressed in (mg/mL). The functional dependence of outcome measures (C_HPHC_, f_HPHC_, C_TPM_, and Y_TPM_) on the combined factors of user topography behavior and product characteristics has not been mechanistically studied and warrants further investigation. The variety of test protocols and outcome measures reported in the literature may simply reflect various laboratory capabilities. Nonetheless, the lack of standardization has made it difficult to compare products across studies or make inferences about the impact of product characteristics on emissions.

The FDA 2016 draft guidance for Pre-Market Tobacco Application, PMTA, for ENDS (81 FR 28781) suggests manufacturers consider the chemical and physical identity and quantitative levels of aerosol emissions under the range of operating conditions and use patterns within which consumers are likely to use the new tobacco product. Previous protocols for combustible cigarettes, influenced by the tobacco industry, resulted in inaccurate emissions that did not represent exposure under actual use conditions (37). The FDA recognizes the influence of topography on emissions, and suggests that topography be considered when assessing substantial equivalence of tobacco products (76 FR 789). Yet product-specific topography data are still lacking and no systematic study has been done to determine appropriate puffing protocols to generate accurate emissions for subsequent chemical constituent analysis. In the absence of studies which characterize the range of user behavior associated with various products, emissions characteristics must be determined for the full range of operating conditions (flow rates and puff durations). Though many studies have investigated aerosol emissions from ENDS over a variety of conditions, none have presented a comparative evaluation of the effective operating envelope of ENDS. This paper addresses this gap by introducing a robust method for empirically quantifying the operating envelope and presents data for thirteen pod- and pen-style ENDS with both button and flow-activated power.

The operating envelope of an ENDS is bounded by the Minimum and Maximum Aerosolization Flowrate (MinAF, MaxAF), and the Minimum and Maximum Aerosolization Duration (MinAD, MaxAD). The MinAF is the puff flow rate above which the ENDS coil consistently activates on every puff and generates TPM yield per puff above the limit of quantitation measurable by the analytical balance. The MaxAF is the puff flow rate below which the ENDS aerosolizes E-Liquid and above which E-Liquid aspiration onset is observed. The MinAD is the puff duration above which the ENDS coil consistently activates on every puff and generates TPM yield per puff above the limit of quantitation. The MaxAD is the puff duration above which no incremental TPM is generated, most likely because the coil has been deactivated by the ENDS power control unit. In the absence of natural environment topography to inform protocols for machine-generated puffing profiles, characterizing products over the entire operating envelope describes the full range of exposure possible for a given ENDS.

Selection of devices to study was based on their relevance in the current ENDS market, product attributes, operating parameters of devices, and manufacturers of ENDS products. We chose to study popular products on the US ENDS market, with a focus on popular products with ohm/sub-ohm coils and “tobacco” flavor e-liquid options. Priority was given to devices with disposable (non-refillable) tanks and fixed (non-user adjustable) power. We selected products from a variety of manufacturers.

In general, the market has trended toward more widespread use of “pod style” devices, an increase in customizability in “mod style” devices, and a trend away from “pen style” ENDs for nicotine use. Additionally, disposable devices that visually resemble pod style products, sometimes referred to as “smoke bars,” have become popular throughout 2020. Meanwhile, pen style ENDS are still relevant for users of Cannabidoil (CBD) and tetrahydrocannabinol (THC) containing e-liquids and oils.

The majority of ENDS product literature refers to the power control unit (PCU) with the benign title “battery,” obfuscating the fact that the PCU often contains sophisticated power management and control logic. Some e-cigarettes have incorporated 510 threads (10 male threads at 0.5 mm pitch with a diameter of 7 mm, aka M7 × 0.5) onto their PCUs to make them compatible with 510 reservoirs. These 510 reservoirs seem to be a product of choice, alongside pods, for mid-chain triglyceride (MCT) solvent e-liquids containing CBD and THC. Therefore, priority was put on choosing pen style products that use a 510 thread. Keyword searches such as “510 batteries” and “510 cartridges” returned ENDS-relevant results for “pen style” devices. While pen style products are not fully customizable, it was observed that some PCUs often offer approximately three discrete voltage settings which users can vary by pushing the activation button in a specific way (e.g., triple click). Devices enabling user adjustable power were not selected for this study. Some ENDS reservoirs are sold with the heating coil integrated into the reservoir, often marketed as with the name “pods” or “cartridges,” while other ENDS reservoirs permit the user to interchange the heating coil. This study focuses primarily on products with reservoirs having integrated heating coils. The ENDS PCU, reservoir and heating coil may each contribute in novel ways to the emissions generated by the device. Thus, it is important to accurately characterize each ENDS product tested to permit meaningful comparisons between products.

The primary objective of this study was to demonstrate an accurate, unbiased method for quantifying the effective operating envelope of ENDS in terms of four parameters: the minimum and maximum aerosolization puff flow rate and duration, denoted MinAF, MaxAF, MinAd, and MaxAd, respectively. The second objective is to report ENDS packaging and product characteristics and descriptive statistics of the nominal coil resistance, R_coil_, and nicotine mass ratio, f_Nic_, expressed as mg of nicotine per mg of aerosolized TPM and un-puffed E-Liquid for each ENDS studied. These product characteristics are proposed for PMTA reporting under 81 FR 28781.

## Materials and Methods

### Test Specimens

**Figure 1:**
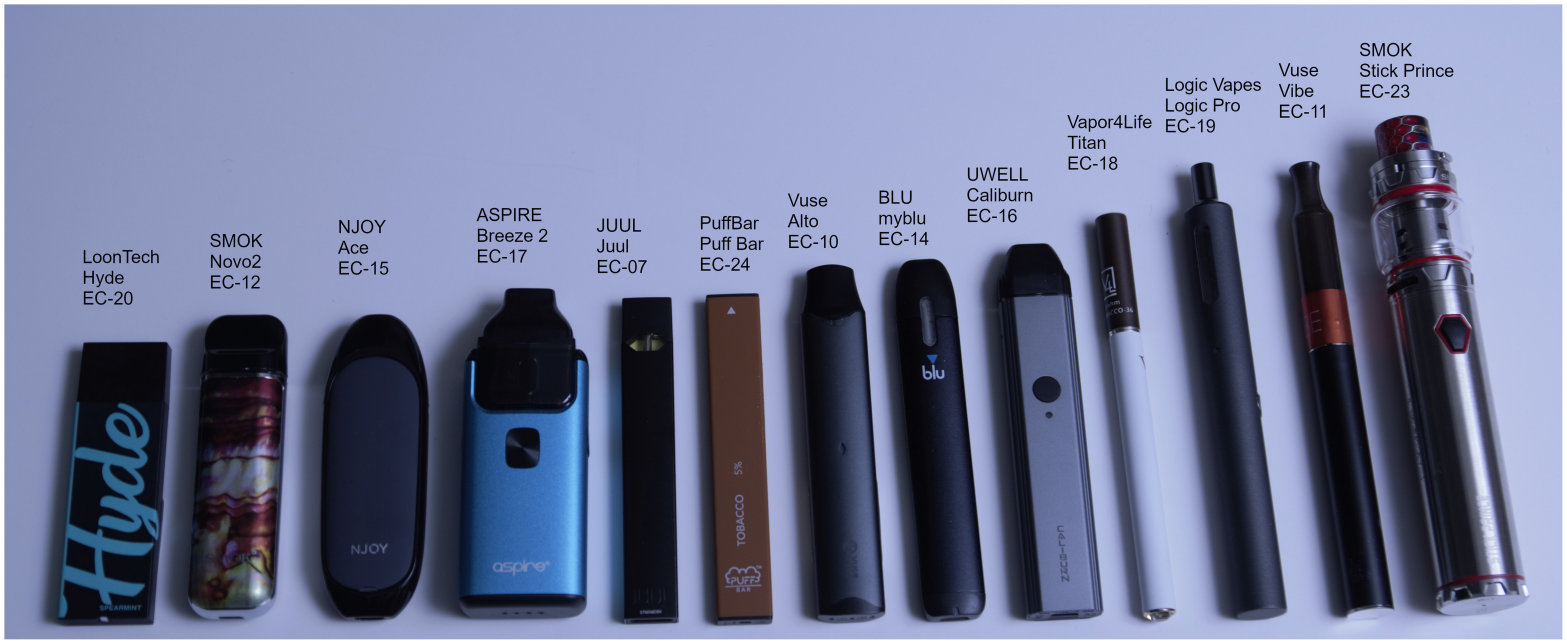
Image of the thirteen ENDS products tested for this investigation.

A summary of the ENDS assessed herein in presented in Table 1. Thirteen unique ENDS products were chosen for this study, including seven popular pod style, four pen-style and two disposable devices as illustrated in Figure 1. Test specimens of each product were obtained from a variety of sources, including the manufacturer's website (M), on-line third party distributors (O), and local retail stores and vape/smoke shops (R). For devices that used pre-filled pods or tanks, the reservoirs were generally purchased at the same time as the PCUs. When additional pre-filled reservoirs were required, they were purchased via the same channel as the original ENDS purchase. For ENDS devices that employed refillable reservoirs, a common e-liquid from the same lot was used, which was purchased over the internet from an e-liquid distributor. All products were purchased between July, 2018 and December, 2020. A New York State law prohibiting on-line and mail-order sales of electronic cigarettes and e-liquid went into effect on July 1, 2020, and all product purchases were made from retail establishments after that date. The source(s) used to purchase the products are provided, along with the country of manufacture. Investigation of company websites and marketing materials was used to identify associated parent companies and/or tobacco-company affiliates of the ENDS manufacturer.

**Table 1:** Test specimens used in screening trials for this study.

**ENDS manufacturer**	**JUUL Labs**	**VUSE**	**SMOK**	**Blu**	**NJOY**	**Uwell**	**Aspire**	**Vapor4Life**	**Logic Vapes**	**Loontech**	**VUSE**	**SMOK**	**Puff bar**
ENDS Model	JUUL	Alto	Novo 2	myblu	Ace	Caliburn	Breeze 2	Titan	Logic Pro	Hyde	Vibe	Smok Stick	Puff Bar
ENDS Style	Pod	Pod	Pod	Pod	Pod	Pod	Pod	Pen	Pen	Single use	Pen	Pen	Single use
**Features**													
Rechargeable PCU	X	X	X	X	X	X	X	X	X	X	X	X	
Refillable reservoir			X			X	X					X	
Replaceable coil			X				X					X	
Adjustable power			By Coil				By Coil					By Coil	
Actuation type	Puff	Puff	Puff	Puff	Puff	Button	Button and Puff	Puff	Button	Puff	Puff	Button	Puff
**Size and shape characteristics**													
PCU form factor	Rect.	Rect.	Rect.	Rect.	Ovoid	Rect.	Rect.	Cyl.	Cyl.	Rect.	Cyl.	Cyl.	Rect.
Reservoir capacity (mL)	0.7	1.8	2	1.5	1.9	2	3	1	1.5	1.8	1.9	8	1.3
Assembled dimensions (mm)Axial × lateral × vertical	94.7 × 15.1 × 6.9	104.6 × 19.1 × 10.6	88.5 × 24.2 × 14.5	106.7 × 18 × 9.5	88.3 × 29.8 OD × 13.5 OD	109.9 × 21.2 × 11.8	94.5 × 35 × 20	121.7 × 9.3 OD	136.6 × 14.1 OD	79.6 × 24.7 × 7.6	136.8 × 13 OD	146 × 24.4 OD	96.6 × 15.6 × 6.5
**Manufacturer reported ENDS characteristics**													
Mfg stated battery chemistry	NR	LiPo	NR	NR	NR	NR	NR	NR	LiOn	LiOn	LiOn	NR	NR
Mfg stated battery capacity (mAHr)	NR	350	800	350	NR	520	1,000	300	650	380	600	3,000	350
Mfg stated coil resistance (ohm)	NR	NR	1.0, 1.4	1.3	NR	1.4	0.6, 1.0	2.3	2.3	NR	NR	0.17	NR
**Mfg** stated power (watt)	NR	NR	6–25 W	10.5 W	NR	11 W Max	NR	NR	3.70 V and 2.3 Ohms	NR	NR	30–70 W	NR
**Manufacturer reported eliquid characteristics**													
Eliquid manufacturer	JUUL Labs	VUSE	Mad Hatter Juice	Blu	NJOY	Mad Hatter Juice	Mad Hatter Juice	Vapor4Life	Logic	Hyde	VUSE	Mad Hatter Juice	Puff Bar
Eliquid branded flavor name	Virginia Tobacco	Original	Classic Tobacco	Classic Tobacco	Classic Tobacco	Classic Tobacco	Classic Tobacco	Wowbacco	Tobacco	Spear Mint	Original	Classic Tobacco	Tobacco
Eliquid branded nicotine concentration	5%	5%	50 mg/5% by vol.	2.40%	5% by wt.	50 mg / 5% by vol.	50 mg / 5% by vol.	3.60%	20 mg/ml	50 mg	3%	50 mg / 5% by vol.	5%
**Purchasing information**													
Place of purchase	M, R	O, R	M	M	M	O	M	M	O	O	R	O	M
Country of manufacturer	China (PCU) USA (Res)	China (PCU)USA (Res)	China	China	China (PCU) USA (Res)	China	China	NR	China	China	China (PCU) USA (Res)	China	China
Parent company	JUUL Labs; Altria	RJRVC; Reynolds America	Shenzhen IVPS Technology Co.	Imperial Brands; Fontem US	NJOY LLC	Shenzhen Uwell Technology Co.	Shenzhen Eigate Technology Co.	Vapor4Life	Logic Technology Development, LLC	Loontech	RJRVC; Reynolds America	Shenzhen IVPS Technology Co.	NR

*M, Manufacturer's website; O, Online distributor; R, Local retail shop; NR, Not Reported; LiPo, Lithium Polymer; LiOn, Lithium Ion; Rect, Rectilinear; Cyl, Cylindrical*.

Each ENDS PCU was documented as it was unpackaged, to observe all labeling on the outer and inner packaging as received from the vendor. The product manufacturer was noted for each device, and ENDS “Product Model” is used as a legend key for subsequent presentation of results. Several attributes of each device were noted, including whether the PCU was rechargeable, the general geometric form factor (shape) of the PCU, and if its e-liquid reservoir was refillable. Most devices studied herein had a coil integrated with the reservoir, while three products permitted user replacement of the coil in the reservoir. The packaging and user instructions were evaluated to determine if each device was flow rate activated, “puff,” or manually activated, “button.” None of the products tested permitted the user to make adjustments to the power, except for the Aspire Breeze 2, SMOK Novo 2, and SMOK Stick which permit users to replace the coil in the reservoir and thereby influence the power dissipated in the coil.

We measured the assembled dimensions of the ENDS PCU plus reservoir in their nominal configuration as intended for use. The volumetric capacity of the reservoir as stated by the manufacturer was recorded, along with the name of the manufacturer of the e-liquid used for each product test. In those cases when the manufacturer provided non-refillable reservoirs, we elected to use e-liquid product from the same manufacturer, marketed for sale with the PCU, and chose the “tobacco” flavored e-liquid. All products which provided refillable reservoirs were operated with a common lot of bulk “classic tobacco” e-liquid manufactured by Mad Hatter Juice. We observed non-uniformity in the units employed to report nicotine concentration of e-liquid, and report the values as observed on the product packaging.

We observed the majority of product packages did not report the battery chemistry, battery capacity, coil resistance, or operating power of the product, and there was non-uniformity in reporting these characteristics between product manufacturers and models. Any data not reported on the external or internal packaging is indicated as “Not Reported (NR)” in Table 1.

Each ENDS Power Control Unit (PCU) and ENDS Reservoir (pod or tank with integrated coil and wick) to be tested was marked with a unique identification number and QR code assigned by the lab. These unique identifiers were scanned prior to each measurement and emissions trial and recorded by data logging scripts. All data measurements and analysis results are traceable by these unique identifiers.

### Gravimetric Test Method

The analytical balance used for this study was a Mettler Toledo Model Number AE240-1 S/N J65956 with a manufacturer reported readability of 0.1 (mg), approximate accuracy of 0.4 (mg) and full scale range of 200 (grams) with a linearity of ± 0.02 (mg) mounted on a heavy work bench to minimize vibration effects. The analytical balance was used to measure the mass of each ENDS reservoir and filter pad “before” and “after” each trial. The decrease in the mass of the ENDS reservoir is one measure of the Total Particulate Matter (TPM) generated by the ENDS via aerosolization while the increase in the mass of the filter pad is a measure of the TPM delivered to the user over the same time interval. When the ENDS Yield (mass decrease) is nearly equal to the Pad Yield (mass increase) there is high confidence that minimal TPM deposition has occurred between the ENDS device and the filter pad in the flow path of the emissions test system.

Sample loading and unloading was done carefully to avoid disturbing the balance, and each sample was positioned near the center of the sample pan, with gentle opening and closing of the balance doors. The analytical balance was maintained at room temperature in the lab, and located in a corner away from drafts and room air ducting. The analytical balance was routinely turned on and allowed to warm up for at least 1 h before taking measurements. Prior to each test series, the analytical balance was confirmed to be level using the bubble level built into the instrument. The accuracy of the balance was verified with its internal standard prior to the beginning of each measurement session. The balance was tared to assure a reading of 0.0000 (gram) prior to placing a sample on the measurement pan.

The limit of detection (LoD) was assumed equal to the gravimetric instrument accuracy at the low end of the measurement range, LoD = 0.4 (mg). The limit of quantitation (LoQ) (38) was set to five times the detection limit, LoQ = 5 LoD = 2.0 (mg). The LoQ was then divided by the number of puffs per each trial (typically 50 puffs) to establish the Y_TPM_ = 0.04 (mg/puff) limit. The relative mass error, ΔM (%), was computed for each observation in the “variable flow rate” series of trials, as the relative difference between the decrease in the mass of the ENDS device, ΔENDS (mg) and the increase in the mass of the filter pad, ΔPad (mg). A large relative mass error is indicative of deposition of aerosol between the exit plane of the ENDS and the surface of the filter pad.

### Coil Resistance Test Method

The effective resistance of the coil and reservoir to PCU connection was measured using a four-wire constant current resistance measurement method as introduced in (39, 40). Custom fixtures were developed for several ENDS products, while hand held measurements were made for the remainder. The two single use ENDS, Hyde and Puff Bar, were destructively opened in order to access their coils' terminals for resistance readings. The coil resistance of each reservoir was measured using four-wire leads connected to a Keysight Model 34465A digital multimeter connected to a data logging computer running a data sampling script. The script was used to read and report the mean and standard deviation of 10 sequential readings of the same reservoir and coil/heating element taken at ~1 s intervals, to monitor stability of the resistance readings. The mean value for each measured reservoir/coil assembly is recorded and assessed to describe the inter-coil variation observed by product.

### Emissions Screening Protocol

Emissions were machine-generated using two sets of puffing profiles: “variable flow rate set” and “variable duration set.” The number of different durations and flow rates run in each set depended on the individual behavior of the product. There were at least 10 profiles run in the variable flow rate set, and each profile had nominally 50 homogeneous square-wave puffs of 3.5 (s) and flow rates ranging from nominally 10 (mL/s) up to 100 (mL/s) for the different profiles in the set. Similarly, there were at least 10 profiles run in the variable duration set, and each profile had nominally 50 homogeneous square-wave puffs of 30 (mL/s), and puff durations ranging from nominally 0.5 to 10 s for the different profiles in the set. All profiles had a nominal puff period of 30 s.

At shorter puff durations or lower flow rates, some products did not activate, and the operator had discretion to conduct additional trials to narrow in on the minimum puff duration or minimum flow rate at which the ENDS began to generate measurable TPM. At higher puff durations or higher flow rates, the operator would limit the number of puffs in the profile to <50 puffs, to ensure the coil remained supplied with e-liquid throughout. For example, one high powered ENDS, when operated for long duration puffs, consumed liquid in the reservoir over only 20 puffs. In those cases, the operator adjusted the series of trials to achieve nominally 50 puffs per flow condition while ensuring that no single emissions profile exhausted the liquid supply or over-loaded the filter pad. For some products, particularly with higher puff flow rates, the operator would need to adjust the range of flow rates studied when significant deposition was visually observed in the tubing of the puffing machine between the exit plane of the ENDS and the entrance to the filter pad.

All emissions tests were conducted using the PES-1 system, previously described (41), which is a computer-controlled programmable puffing and emissions capture machine, consisting of a flow controller connected to a vacuum tank. Puff flow rate was controlled using a proportioning valve with command signals from a closed-loop feedback controller. For button activated devices, an actuator commanded by the computer pushed the PCU activation button of button-activated PCUs at the start of each puff, and released the button at the end of a puff. Flow rate measurements were made using an Alicat Scientific M-50SLPM-D-30PSIA/5M calibrated flow meter sampled by the computer at a rate of 100 Hz. Tank vacuum pressure was fixed at 37.4 (kPa) and was maintained by a vacuum pump and a proportioning valve. The PES-1 system can generate puffs between 5 and 150 (mL/s) with puff duration and inter-puff gap as small as 0.2 (s) (41). There is no maximum limit for puff duration and inter-puff gap. The command profile was specified to the system as a flow rate time series. All topography parameters (puff flow rate, duration, interval) were reported “as measured” in order to ensure that any inaccuracies in the ability of the emissions system to follow any particular command profile do not introduce error (41). Time-stamped flow rate measurements were stored in a csv file along with information of the product used and the experimental setup parameters. Vapor phase emissions were collected on Cambridge filter pads.

### Analytical Chemistry Methods

NMR analysis was conducted on un-puffed E-Liquid samples taken from each product tested to determine the proportion of propylene glycol to the glycerin which formed the solvent base for the liquid. The instrument used for NMR was a Bruker Advance III 500 MHz NMR (Billerica MA). Approximately 10 mg of an e-liquid was added to an NMR tube followed by 600 μL of D2O and a typical NMR spectrum was obtained. After NMR spectra were obtained the spectra were processed using KnowItAllTM spectral processing software (Wiley). For each sample, the water peak was centered at 4.79 ppm and baseline was corrected to ensure proper integration. Peaks for propylene glycol (3.38–3.47 ppm, 1H) and glycerol (3.605–3.675 ppm, 2H) were integrated and molar ratios of each were determined. The integration ranged varied for e-liquids with acid added (known as salted e-liquids) as pH will slightly shift NMR peaks. Volume and mass ratios were calculated for each solution using known density and molar mass of each. To confirm the validity of the method, mixtures of propylene glycol and glycerol (mass ratios: 0:100, 25:75, 50:50, 75:25, 100:0) were prepared and analyzed using the described method. In all cases, correct ratios were confirmed for each test mixture.

GC-MS analysis was conducted to determine the mass ratio of nicotine to total particulate matter from the condensed aerosol captured on the filter pad during emissions trials. Emissions trials above the mass limit of detection were analyzed. Mass of nicotine in the aerosol was determined in the same manner as previously reported (42). In brief, pads used to collect aerosol were spiked with quinoline as an internal standard, submerged in methanol, shaken to break up the pad and filtered prior to quantification. Nicotine concentration was determined by GC-MS (Shimadzu QP2010 GCMS with an AOC-20s autosampler). Each sample was run in triplicate to ensure accurate results. The mass fraction of nicotine in the aerosol (f_NIC_) was calculated as the ratio of the mass of nicotine found on the pad to the total mass of particulate matter deposited.

### Determination of Operating Envelope

Three figures were generated for each ENDS model and used collectively to assess the effective operating envelope of each product (not shown). The emissions testing of each model may require multiple devices (ENDS PCUs and Reservoirs) as reported in the Results. All devices associated with a particular ENDS model were analyzed together. The figures included a scatter plot of (1) pad yield per-puff Y_TPM_ (mg/puff) vs. mean puff flow rate q (mL/s) for the “variable flow rate set” of conditions, (2) Y_TPM_ (mg/puff) vs. mean puff duration d (s) for the “variable duration set” of conditions, and (3) relative gravimetric error ΔM (%) vs. q (mL/s) for the “variable flow rate set” of conditions.

Each figure was annotated with notes recorded by the operator during trials. For example, the operator recorded when an LED indicator behaved in a different manner. Some ENDS devices, for example, documented the LED would change color when the battery dropped below a predefined voltage or when the maximum puff duration was exceeded. Observations of the LED were then compared with other quantifiable characteristics, such as changes in TPM pad yield per puff. The operator noted if any bubbles appeared to be generated in the ENDS Reservoir, if droplets were evident in the connection between the exit plane of the ENDS device and the surface of the filter pad, or if discrete droplets or gravity distribution of deposition pattern were evident on the filter pad. Likewise, the operator noted if there was a significant increase in the coil resistance between the “before” and “after” resistance measurements when using the fixture 4 wire resistance measurement method (39, 40), and if so, would retire that reservoir from further testing to decrease the likelihood of using a failed coil in further trials. The operator noted whether each puff-activated ENDS appeared to consistently activate for every puff in the multi-puff sequence, or if the ENDS device operated unreliably.

After all three scatter plots were generated and annotated for each ENDS, the analyst interpreted the yield results in the context of the emission operator's notes. The analyst determined four parameters to characterize the effective operating envelope of each ENDS: (1) the minimum aerosolization flow rate (MinAF), (2) the maximum aerosolization flow rate (MaxAF), (3) the minimum aerosolization duration (MinAD), and (4) the maximum aerosolization duration (MaxAD).

The MinAF was defined as the lowest puff flow rate at which the ENDS device consistently activated and generated aerosol, while the puff duration was held fixed at nominally 3.5 sec. The MinAF simultaneously (1) generated yield above the per-puff LoQ, Y_TPM_ ≥ 0.04 (mg/puff) for the “variable flow rate set” of conditions, (2) exhibited a relative gravimetric error, ΔM ≈ ≤ 10 (%), and (3) appeared to consistently activate the ENDS coil based on operator observations. The error bound on the MinAF were taken to be the difference between trial conditions wherein constraints 1 through 3 were and were not consistently satisfied.

The MaxAF were defined as the lowest flow rate at which there was visual and gravimetric evidence of aspiration, while the puff duration was held fixed at nominally 3.5 s. The MaxAF simultaneously (1) exhibited a sudden sharp increase in the slope of the Y_TPM_ vs. q curve, (2) exhibited a relative gravimetric error, ΔM > 10 (%), and (3) exhibited evidence of liquid suction in addition to or in place of aerosolization as reflected by operator observations and photographs. The error bound on the MaxAF were taken to be the difference between flow conditions wherein constraints 1 through 3 were and were not consistently satisfied. If the MaxAF could not be determined definitively for the conditions tested, the maximum flow rate tested was recorded.

The MinAD were defined as the lowest puff duration at which the ENDS device consistently activated and generated aerosol while the nominal puff flow rate was held fixed at ~30 mL/s. The MinAD simultaneously (1) generated yield above the per-puff LoQ, Y_TPM_ ≥ 0.04 (mg/puff) curve, (2) exhibited a relative gravimetric error, ΔM ≈ ≤ 10 (%), and (3) appeared to consistently activate the ENDS coil based on operator observations. The error bound on the MinAD were taken to be the difference between duration conditions wherein constraint 1 through 3 were and were not consistently satisfied.

The MaxAD were defined as the upper time limit above which the ENDS no longer provided power to the coil. Many, not all, ENDS manufacturers cut off the current provided to the coil after some manufacturer-determined time limit, and this feature is not reported by most manufacturers. The MaxAD simultaneously (1) exhibited a distinct flattening of the Y_TPM_ vs. d curve, (2) exhibited a relative gravimetric error, ΔM ≈ ≤ 10 (%), and (3) appeared consistent with operator visual and audible observations of ENDS behavior. The error bound on the MaxAD were taken to be the difference between conditions wherein constraints 1 through 3 were and were not consistently satisfied. If the MaxAD could not be determined definitely for the conditions tested, the maximum duration tested was recorded.

## Results

**Figure 2:**
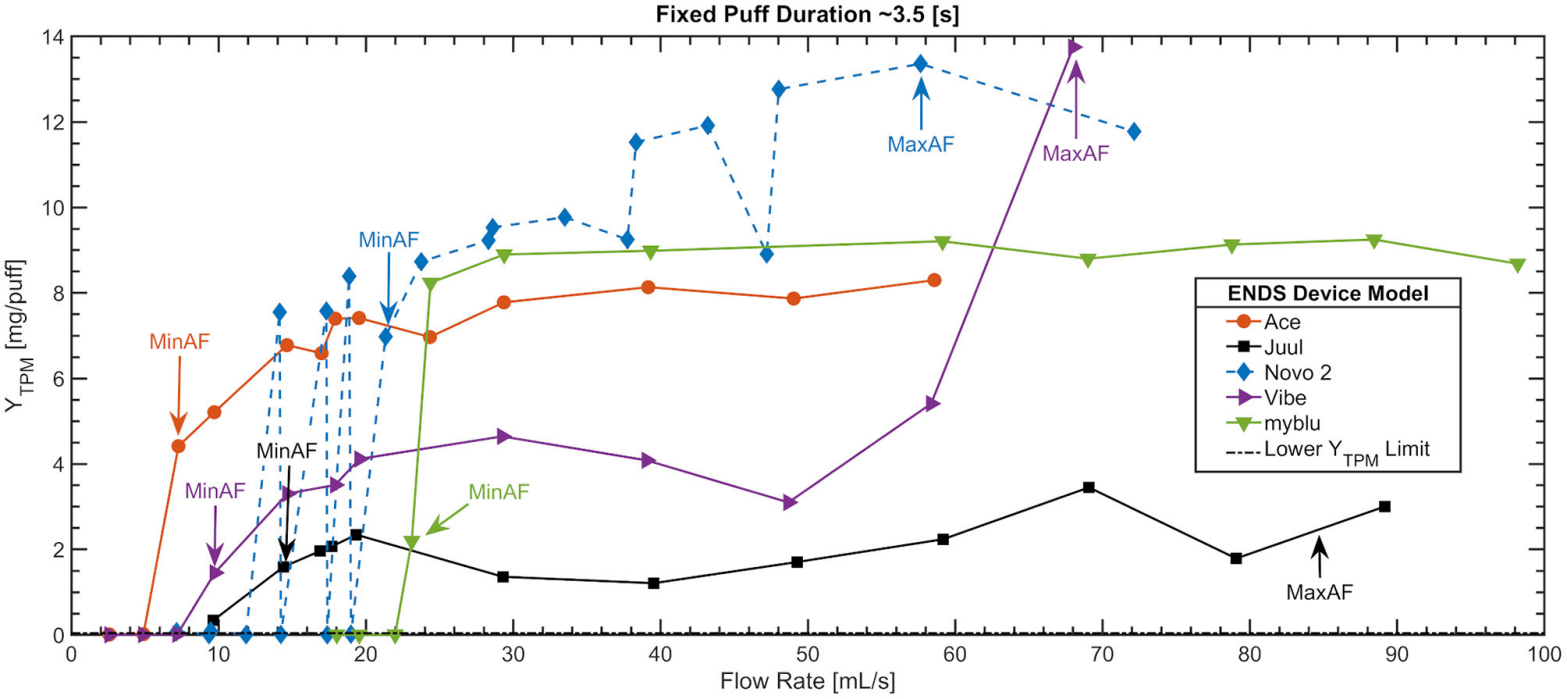
Minimum and maximum activation flow rate, MinAF and MaxAF, for 5 of the 13 ENDS devices tested. Shown is the mean TPM yield per puff for each flow rate condition, based on nominally 50 puffs per flow rate conditions. Puffs were square-wave and nominally 3.5 s in duration, with 30 s puff period.

Figure 2 shows a scatter plot of TPM pad yield per puff deposited on the filter pad as a function of flow rate for five exemplar ENDS products: JUUL LABS Juul and BLU myBlu (both puff-activated non-refillable pod-style), NJOY Ace (button-activated non-refillable pod-style), SMOK Novo 2 (button-activated pod-style with refillable reservoir and user-replaceable coil), and VUSE Vibe (puff-activated non-refillable pen-style). Experimental results for all thirteen products are presented in Supplementary Material. The data are reported as a function of the actual mean flow rate achieved by the emissions system (41) across nominally 50 puffs per trial. The PES command puff duration was 3.5 (s) and the actual puff duration achieved by the emissions system had a mean of 3.29 (St. Dev. 0.37) (s). The LoQ Lower Y_TPM_ limit of 0.04 (mg/puff) is represented as a horizontal broken line. Lines are used to connect the markers as a visual aid and are not intended to be indicative of a curve. Selected operator and analyst annotations regarding the MinAF and MaxAF are presented on the figure.

Consider the button activated Ace device which generated low TPM at flow rates of 2.6 and 4.9 (mL/s) with a noticeable increase at 7.3 (mL/s). The MinAF for the Ace was declared to be 6 ± 1.2 (mL/s). Similarly, the MinAF was declared to be 9.75 ± 3.5, 12 ± 2.4, and 23 ± 1.2 (mL/s) for the Vibe, Juul, and myBlu, respectively. The Novo 2 exhibited erratic behavior between 12 and 18 (mL/s). Operator notes indicated the device failed to “fire consistently” for every puff, and trials also exhibited relatively large gravimetric error, shown in a subsequent figure. The Novo 2 appeared to be fully activated at flow rates above 24 (mL/s) and essentially inactive at flow rates below 22 (mL/s). Thus, the MinAF for the Novo 2 was declared to be 21.3 ± 2.5 (mL/s) with the broad error bar indicating the observed variability in the ENDS performance. The maximum pad yield per puff was Y_TPM, Max_ ≈13 (mg/puff) for the Novo 2 and the lowest was Y_TPM, Max_ ≈ 2 (mg/puff) for the Juul, which was also the minimum value of Y_TPM, Max_ across the 13 products studied. Conversely, the Stick Prince, presented in Supplementary Material exhibited the highest pad yield per puff of Y_TPM, Max_ ≈ 50 (mg/puff) within the normal operating range of all devices tested (excluding cases where e-liquid was clearly aspirated into the flow path). Figure 2 illustrates the relatively erratic yield response of the Novo 2, while the Vibe appeared quite stable until 45 (mL/s), with an unusual response at 60 (mL/s), and then evidence of aspiration was observed officially at 70 (mL/s). The myBlu product exhibited the most uniform TPM yield per puff across the range of flow rates of all thirteen products studied here. Both the emissions stability and value of Y_TPM, Max_ are potentially significant regulatory outcome measures. Of the thirteen products tested here at a nominal puff duration of 3.5 (s), for example, the Stick Prince delivered the highest Y_TPM, Max_ ≈ 50 (mg/puff) while the Juul delivered the lowest Y_TPM, Max_ ≈ 2 (mg/puff) – a ratio of 25:1. Clearly, some ENDS are capable of delivering far more TPM to the mouth of a user within the product's normal operating envelope.

**Figure 3:**
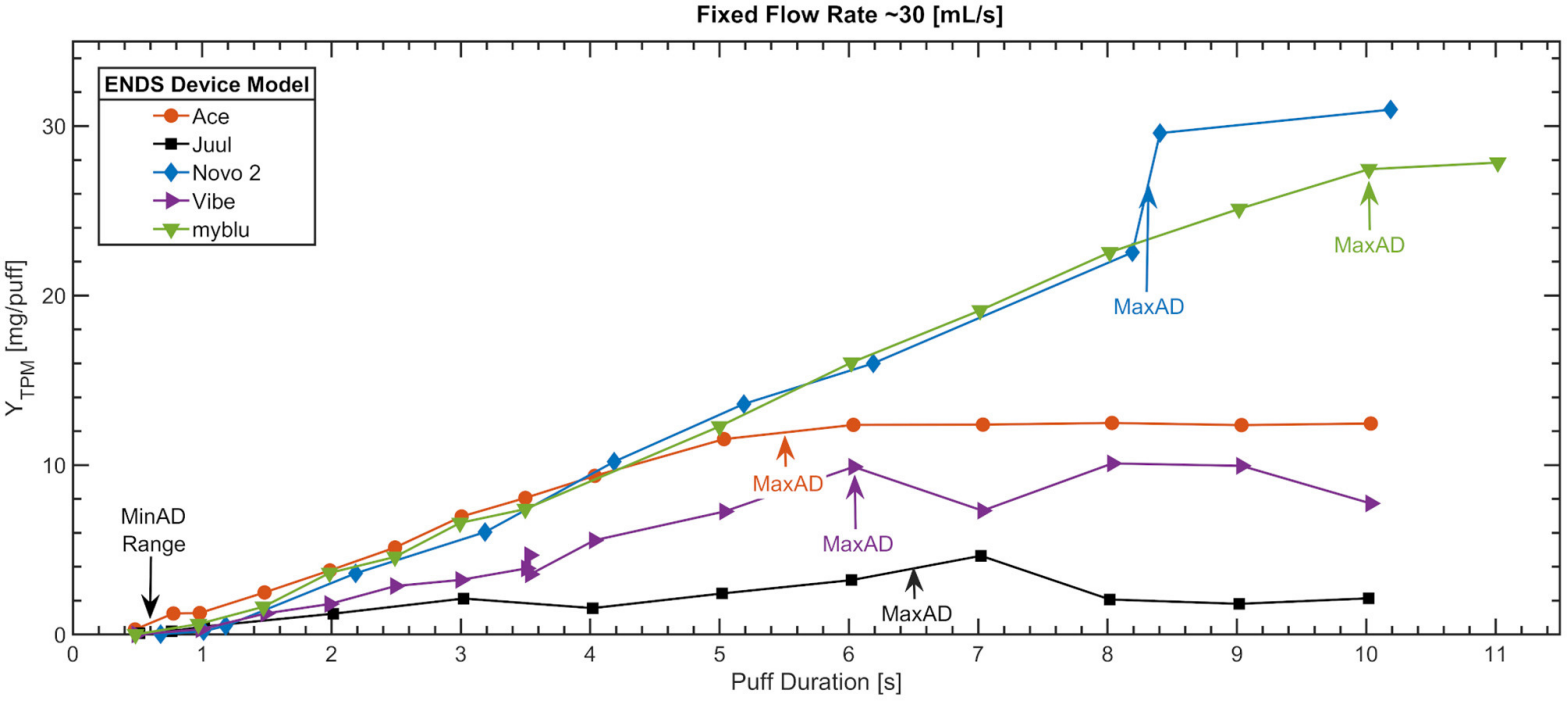
Minimum and maximum activation duration, MinAD and MaxAD, for 5 of the 13 ENDS devices tested. Shown is the mean TPM yield per puff for each duration condition, based on nominally 50 puffs per flow rate conditions. Puffs were square-wave and nominally 30 ml/s, with 30 s period.

Figure 3 shows a scatter plot of TPM yield per puff deposited on the filter pad as a function of duration for the same five ENDS presented in Figure 2. Each emissions trial result is represented by a single marker; lines are a visual aid only. The data are reported as a function of the actual mean puff duration achieved by the emissions system (41) across nominally 50 puffs per trial. The command puff flow rate was 30 (mL/s) for all conditions presented in this figure, while the measured puff flow rate had a mean of 28.5 (St. Dev. 1.5) (mL/s). The LoQ Lower Y_TPM_ limit of 0.04 (mg/puff) was first exceeded when the ENDS mean duration ranged between 0.5 and 0.65 (s) for the five ENDS illustrated, denoted as the range of MinAD.

The five PCUs are useful to illustrate unique features evident across the family of thirteen devices tested. First, we observe the myBlu response, which exhibits a linear increase in pad yield per puff (at this fixed flow rate) until a duration of 10 (s) is achieved, at which point the yield curve flattens out. This is consistent with operator observations that the PCU de-energized after approximately MaxAD ≈ 10 (s) puff duration. The Ace PCU curve exhibited similar response, with the PCU de-energizing at approximately MaxAD ≈ 5.5 (s) as supported by both operator observation and manufacturer documentation. The Vibe PCU exhibited similar response; the operator notes indicate a cut-off at approximately MaxAD ≈ 6 (s), with greater variability in emissions yield from Vibe relative to the other ENDS. The Novo 2 PCU exhibited a fundamentally different response. The expected linear increase of yield with duration (at fixed flow rate) was observed until 8 (s), but then a dramatic increase in yield was observed. Operator notes taken during the last two trials indicated visual evidence of liquid being suctioned from the ENDS reservoir, and deposited between the exit plane of the ENDS and the entrance to the filter pad surface [a distance of <1 (cm)], which we declare as the onset of aspiration. The Juul ENDS exhibited a comparatively constant pad yield per puff as a function of duration (at fixed flow rate) though some linearly increasing trend is implied between 4 and 7 (s).

**Figure 4:**
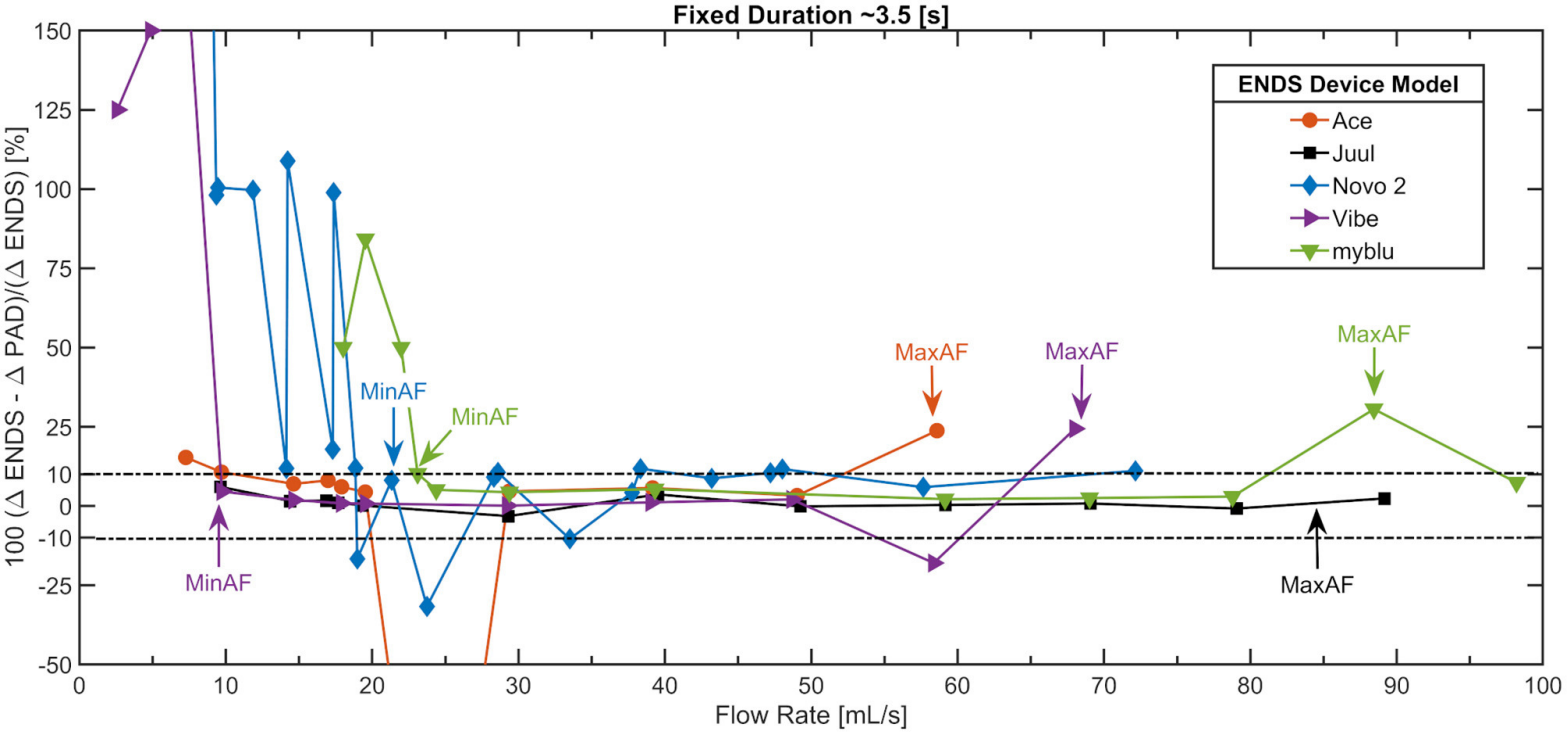
Relative gravimetric measurement error related to operator observed aspiration during the “Variable Flow Rate set” of conditions shown in Figure 1 for 5 of the 13 ENDS. A relative mass error of ΔM > ± 10% at flow rates exceeding the activation flow rate was consistently associated with visual evidence of liquid aspiration. Puffs were square-wave with nominal puff duration of 3.5 (s) and 30 s puff period.

Figure 4 shows a scatter plot of the gravimetric measurement consistency as a function of flow rate arising from the same conditions presented in Figure 2. The vertical axis is relative percent error, ΔM, computed as the total mass decrease observed in the ENDS compared to the total mass increase observed on the filter PAD for each condition. The MinAF determined by Figure 2 is repeated here for reference. Operator notes associated with onset of liquid aspiration are annotated as an aide to understand corresponding conditions between Figures 2, 4. The large relative errors at low flow rates are associated with the small magnitudes observed and reflect that the data is below the acceptable LoQ. The relative mass error reiterates the results presented in Figure 1 for the Novo 2 which behaves erratically at flow rate below MinAF ≈ 21.3 ± 2.5 (mL/s). All five ENDS illustrated here exhibited visual and/or deposition evidence of liquid aspiration as the flow rate increased. Aspiration onset was evident at flow rates as low MaxAF ≈ 48 (mL/s) and as high as MaxAF ≈ 88 (mL/s) for the thirteen products studied. The Novo 2 exhibited potential aspiration and visibly large droplets even at moderate flow rates. The Juul in Figure 4 exhibited the first visible signs of aspiration (MaxAF criteria 3) between 80 and 90 (mL/s).

The analysis described in the methods and illustrated by Figures 2–4 was applied to experimental data from all 13 ENDS designs. A summary of findings is presented in Table 2. The mean and median f_Nic_ ratios are reported as the average across all flow conditions within the operating envelope of each device. There was insufficient evidence in the results from the screening trials to assess significant variation in f_Nic_ as a function of flow rate or duration. The primary outcome measures for the operating envelope (MinAF, MaxAF, MinAD, MaxAd) are shown in the upper portion of the table. The mean, median and standard deviation effective coil resistance using the four wire resistance measurement method (40), emissions nicotine mass ratio, un-puffed E-Liquid nicotine mass ratio, and solvent Propylene Glycol to Glycerin composition are reported for each product. The number of PCUs and reservoirs used for each device model are presented in Table 2, and ranged from a low of 1 PCU and reservoir up to 8 PCU/Reservoir combinations for disposable ENDS. Comprehensive characterizations, well beyond the screening conditions described herein, require more PCUs and reservoirs; we used 5 PCUs and 41 reservoirs in assessment of the Vuse Alto.

**Table 2:** Nominal operating envelope and emissions characteristics of test specimens used in this study.

**Product: model**	**JUUL**	**Alto**	**Novo 2**	**myblu**	**Ace**	**Caliburn**	**Breeze 2**	**Titan**	**Logic Pro**	**Hyde**	**Vibe**	**Smok Stick**	**Puff Bar**
Eliquid manufacturer	JUUL Labs	VUSE	Mad Hatter Juice	Blu	NJOY	Mad Hatter Juice	Mad Hatter Juice	Vapor4Life	Logic	Hyde	VUSE	Mad Hatter Juice	Puff Bar
**Operating envelope**													
Number of ENDS PCU's tested	1	5	1	1	1	1	1	2	1	8	1	1	5
Number of ENDS reservoirs tested	5	41	1	13	9	1	1	6	8	8	6	1	5
Min AF (mL/s)	12 ± 2.4	15.8 ± 1.1	21.3 ± 2.4	23 ± 1.2	6 ± 1.2	11.8 ± 2.25	2.5 ± 2	12.2 ± 2.5	2.6 ± 2.25	16 ± 1.6	9.75 ± 3.75	2.5 ± 2.5	14 ± 4
Max AF (mL/s)	>85 ± 5	50 ± 2	58 ± 10	88 ± 5	58 ± 5	> 88	~30 @ d > 8 s	> 50	>48	>86	68 ± 5	ADR	>50
Min AD (s)	0.5 ± 0.25	0.85 ± 0.15	0.9 ± 0.15	1 ± 0.25	0.5 ± 0.25	0.5 ± 0.25	1 ± 0.52	1 ± 0.25	0.75 ± 0.25	0.5 ± 0.25	1 ± 0.25	1 ± 0.25	1 ± 0.25
Max AD (s)	6.5 ± 0.5	5.0 ± 0.1	8 ± 0.5	10 ± 0.5	5.5 ± 0.5	>10	10.5 ± 0.25	10 ± 0.25	>10	>10	6 ± 0.5	8 ± 0.25	>3.5
**Emissions and E-liquid characteristics**													
Number of f_Nic_ samples	21	135	13	17	25	27	24	15	27	20	11	38	11
f_Nic_ Mean (-)	0.046^a^	0.0468	0.0503	0.0256	0.055	0.046	0.036	0.0305	0.0211	0.0596	0.0315	0.0496	0.0454
f_Nic_ Median (-)	0.046^a^	0.0464	0.05097	0.025	0.055	0.046	0.039	0.0295	0.0197	0.0607	0.0304	0.0523	0.0486
f_Nic_ Std Dev (-)	0.003^a^	0.0048	0.0038	0.0033	0.0046	0.0037	0.009	0.00296	0.007	0.0041	0.005	0.0058	0.0063
Eliquid nicotine mass ratio (-)	0.052	0.052	0.039	0.02	0.05	0.039	0.039	0.023	0.016	0.0503	0.030	0.039	0.048
Eliquid nicotine mass ratio StDev (-)	0.0005	0.0008	0.0019	0.0012	0.0002	0.0019	0.0019	0.0009	0.0009	0.0017	0.0004	0.0019	NR
Eliquid molar ratio PG:GL	33:67	NR	44:56	42:58	48:52	44:56	44:56	73:27	77:23	NR	23:77	44:56	54:56
Eliquid mass ratio PG:GL	29:71	NR	39:61	38:62	43:57	39:61	39:61	69:31	74:26	NR	20:80	39:61	50:50
Eliquid volume ratio PG:GL	33:67	NR	44:56	42:58	48:52	44:56	44:56	73:27	77:23	NR	23:77	44:56	54:56
**Heating element/coil characteristics**													
R measurement method	F4W	F4W	M4W	F4W	F4W	M4W	M4W	M4W	M4W	M4W	M4W	M4W	M4W
Number of coil R measured	16	17	3	18	8	3	1	4	8	1	6	2	1
Effective coil resistance mean (ohm)	1.633	1.063	1.463	1.416	1.034	1.405	0.631	2.258	2.443	1.61	2.693	0.174	1.688
Effective coil resistance StDev (ohm)	0.033	0.075	0.047	0.017	0.079	0.013	0	0.053	0.077	0	0.018	0.006	0

*NR, Not Reported; F4W, Fixture 4 Wire; M4W, Manual 4 Wire*.

a *These nicotine mass ratios were computed without the use of the internal standard (IS) and simply from calibration curve of nicotine due to an error in the IS reference sample preparation*.

## Discussion

### Limitations and Scope

The results presented herein are for 13 ENDS products of either pen- or pod-style designs, and share a common attribute of no user-adjustable power options (other than swapping coils), and no user adjustable flow path (e.g., variable inlet restrictors). The study investigated both flow- and button-activated coil designs and disposable and refillable reservoirs. The numerical values presented in the results, discussion and conclusion may thus be limited to pen- and pod-style ENDS. The screening method and outcome measures may be broadly applied to a variety of inhaled tobacco products including ENDS, combustibles and heated tobacco products (also referred to as “Heat Not Burn”).

This article has presented a comparative evaluation of the effective operating envelope of thirteen popular ENDS (pod and pen-style) products. Concurrently, the article demonstrated a robust method for empirically quantifying the operating envelope of ENDS products. The method may be used to compare operating envelope and emissions characteristics between ENDS products and may enable data sharing and reproducibility studies between research laboratories.

ENDS products have the ability to limit the maximum coil activation time and hence TPM per puff.

The maximum aerosolization duration, MaxAD, after which the ENDS power control unit de-energizes the coil, varies widely between ENDS products. All thirteen ENDS products permitted puff durations of at least 5 s. Five products energized the coil for puff durations between 5 and 7.5 s, five products between 7.5 and 10 s, and three products continued to energize the coil for more than 10 s (the maximum duration studied in these experiments). There is no doubt that limiting the coil activation duration time, MaxAD, is feasible; this is a product characteristic which may be regulated and has a direct correlation with the per-puff TPM delivered to a user.

### Aerosol Observed Nicotine Mass Ratio Is Comparable to That in Unpuffed E-Liquids

We observed a correlation between the nicotine mass ratio in the un-puffed e-liquid (denoted Eliquid nicotine mass ratio in Table 2) and the mass ratio present in the generated aerosols (denoted f_Nic_ in Table 2), for both the mean (β = 1.068, r = 0.84, R^2^ = 0.700) and the median (β = 1.085, r = 0.86, R^2^ = 0.735). We found the nicotine mass ratio to be largely independent of puff flow rate, duration, and volume by conducting a multi-variate linear regression analysis of f_Nic_ for all 13 products. There was insufficient evidence to support a flow condition dependence of f_Nic_ for eleven products (*p* > 0.05). The Novo 2 exhibited an f_Nic_ slightly dependent on flow rate (β = 0.003, *p* = 0.045) and puff duration (β = 0.023, *p* = 0.049), but not on puff volume (β = −0.0009, *p* = 0.053). The SMOK Stick value of f_Nic_ was slightly associated with puff volume (β = 0.0002, *p* = 0.038) but was not associated with either puff flow rate (β = 0.0007, *p* = 0.073) or duration (β = −0.005, *p* = 0.097). None of the ENDS products selected for this study permitted user-adjustable power settings. It remains an unanswered question, worthy of further investigation, to assess the dependence of f_Nic_ for higher power devices such as box-mod ENDS.

We conclude that, for moderately powered pen-style and pod-style devices, it is a reasonable first-order approximation to assume the mass ratio of nicotine present in the aerosol emissions is similar to the mass ratio of nicotine in the un-puffed E-Liquid. Pagano et al. (42) studied five brands of first generation cig-a-likes and reported the nicotine mass delivered to the pad ranged from 14 to 58% of the nicotine mass in the un-puffed cig-a-like ENDS. Pagano et al. defined this ratio as the nicotine transfer efficiency and suggested a significant mass of nicotine was retained in the cig-a-like wick at its end of life. In this study, we specifically avoided puffing the ENDS until the reservoir was empty. While the Pagano article demonstrated that cig-a-like ENDS inherently retained significant residual nicotine at end-of-product-life, modern pen- and pod-style ENDS can deliver virtually all of the nicotine from the reservoir to the user. A parallel study investigates the relationship between consumption of all E-Liquid in two pod-style ENDS reservoirs and its impact on coil lifetime (43). As power levels increase and coil temperature is permitted to rise, as anticipated for modern sub-ohm box-mod style ENDS, the variability in the saturation temperature of the E-Liquid constituents is likely to invalidate this approximation, and caution should be taken if extrapolating this approximation to other devices and E-Liquids.

### Most ENDS Devices Exhibit E-Liquid Aspiration in Addition to Aerosolization

ENDS are known to produce condensation aerosols which contain submicron particles suspended in vapor and inhalable by the user. However, we observed at high flow rates formation of droplets in the flow path of the emission system and in some cases formation of bubbles within the un-puffed E-Liquid in the ENDS Reservoir. We hypothesize this phenomena, which we have named E-Liquid aspiration, to result from excess suction pressure in the reservoir at high flow rates sufficient to overcome the surface tension of the solvent. This is analogous to sucking liquid droplets through a straw when the container is nearly empty and the distal end of the straw is not submerged. While our visual observations were consistent with fluid transport phenomena, further investigation is warranted. If indeed E-Liquid aspiration was occurring at user-achievable flow rates, this could be a potential poisoning hazard. Al-Delaimy and Sim (44) documented up to 4,000 cases annually of E-Liquid and ENDS poisoning in the USA since 2014. Even if users are not aspirating an entire bolus of E-Liquid, such mechanisms could dramatically increase the yield of TPM, nicotine and other E-Liquid additives in a single puff, as observed by the data, and alter patterns of lung deposition. Aspiration of MCT oils (commonly used in ENDS devices for delivery of CBD and THC active ingredients) in liquid form has potentially significant adverse health implications, particularly in light of public health concerns related to E-cigarette or Vaping Product Use-Associated Lung Injury (EVALI) (45).

### Recommended Product Characteristics for ENDS Regulations

Traditional product characteristics considered for regulation include items such as E-Liquid nicotine concentration and coil resistance. However, such regulations may not achieve the desired public health outcomes. Even if ENDS manufacturers are constrained to a maximum E-Liquid nicotine mass concentration, they are able to adjust numerous product characteristics to achieve a high nicotine yield per puff including: increase the PCU Maximum Aerosolization Duration (MaxAD), decrease the coil resistance, increase the coil voltage or current, increase the coil power duty cycle, decrease the ENDS flow path resistance, or decrease the solvent saturation temperature. All of these adjusted product characteristics may result in potentially adverse unintended public health consequences. In fact, decreasing the nicotine concentration in the E-Liquid, while keeping all other product characteristics fixed, will result in a net increase in TPM exposure for a user who consumes a given mass of nicotine per day. We propose it is more effective to regulate the product characteristics of TPM (Y_TPM_) and nicotine yield per puff (Y_Nic_ = f_Nic_ × Y_TPM_ = f_Nic_ × C_TPM_ × V_Puff_). In the proposed case, manufacturers have free reign to adjust numerous design parameters of their PCUs and E-Liquids, but the end-result outcome measure remains consistently regulated.

### Recommended Standard Manufacturer Packaging Information for ENDS Devices and E-Liquids

We observed variability in descriptions provided to consumers on product packaging between manufacturers. We recommend manufacturers be required to prominently disclose several characteristics of ENDS Power Control Units including: battery chemistry and capacity (mAh), designed flow rate operating range, designed maximum coil activation puff duration, and operating range of root mean square (RMS) power (watts) dissipated in a coil of a stated nominal design coil resistance (ohms). We recommend manufacturers be required to prominently disclose several characteristics of ENDS Reservoirs including: all materials present in the reservoir, solder, wick, coil, and mouthpiece, the reservoir fill volume (mL), the nominal coil resistance (ohm) and coil manufacturing variability expressed as a standard deviation (ohm). We recommend that E-Liquid manufacturers (including E-Liquid sold in disposable reservoirs and refill liquid) be required to prominently display the composition of un-puffed E-Liquid in tabular format listing the solvent components and composition (i.e., Propylene Glycol, Glycerin, water, etc.) (22) and additives including nicotine, menthol, and all other additives on a mass fraction basis, such that the sum of all constituents is unity. This is similar to the nutrition labels familiar to many consumers on food products. These product characteristics (ENDS PCU, Reservoir and E-Liquid) collectively affect the mass concentration and composition of emissions generated by ENDS devices and consumables.

### Recommended Standard Emissions Outcome Measures

No standard emissions outcome measures have previously been agreed upon. We recommend that ENDS manufacturers be required to conduct and report flow condition dependent emissions as an integral aspect of premarket regulatory approval processes. The emissions trials should be conducted over the entire range of operating envelope indicated on their product packaging and consumer information. Manufacturers should be required to report emissions outcome measures at each of several operating conditions (puff flow rate, puff duration and RMS coil power) spanning the product operating envelope. Emissions outcome measures should include at least: Total Particulate Matter (TPM) yield per puff [Y_TPM_, (mg/puff)], TPM mass concentration [C_TPM_, (mg/mL)], and aerosol mass ratio of every constituent listed in the un-puffed E-Liquid [f_constituent_, (mg constituent/mg TPM)]. The nicotine mass ratio was demonstrated for eight different E-Liquids herein as one example of a constituent mass ratio. Use of the emissions outcome measures (Y, C, f) has been demonstrated previously for a variety of products (11, 46) and can be used as input characteristics to an experimentally validated behavior-based yield model (46–51). With addition of this article documenting thirteen ENDS, these outcome measures now provide a basis for future product comparisons.

### Recommended Standard Information for PMTAs

Collectively, these labeled product characteristics and emissions pre-market data serve to document the effective operating range (envelope) of an ENDS product. We propose this documentation be mandated for PMTA reporting under 81 FR 28781. The product labeling information ensures that consumers are well-informed of potential chemical exposure arising from actual product use. The proposed emission outcome measures permit regulators to assess the likelihood of potentially hazardous decomposition products which may be present in the emissions. If the E-Liquid product manufacturers are required to document 100% of the product's mass ratio content and the Reservoir product manufacturers are required to document the materials present in the ENDs reservoir assembly, then the union of these two documents result in a relatively short list of f_constituent_ outcome measures, which can be used to inform regulators of potential decomposition products. That is, if the summation of all f_constituent_ reported in the emissions adds to < 100% of the mass collected during emissions testing, there is a reasonable probability that other compounds may be present in the emissions, thus warranting further regulatory review prior to market approval.

## Conclusions

A standard method for characterizing the operating envelope of ENDS products using four parameters (MinAF, MaxAF, MinAD, and MaxAD) has been presented and demonstrated. The study demonstrated good emissions study practices by thoroughly documenting the ENDS test specimen product characteristics, TPM yield per puff, Y_TPM_, TPM mass concentration, C_TPM_, and descriptive statistics of the nominal coil resistance, R_coil_, and nicotine mass ratio, f_Nic_, expressed as mg of nicotine per mg of TPM.

Three emissions outcome measures (Y, C, f) are recommended for adoption as standard quantities for emissions testing by manufacturers and research laboratories. Recommendations for minimum required product labeling have been proposed for ENDS power control units, reservoirs, coils and E-Liquids. Recommendations for required data to be included in premarket tobacco applications have been proposed.

Further investigation into mechanisms of E-Liquid aspiration is needed to inform potential regulations.

## Data Availability Statement

The original contributions presented in the study are included in the article/Supplementary Material, further inquiries can be directed to the corresponding author/s.

## Author Contributions

EH, RR, and NE: conceptualization and funding acquisition. GD, NE, and EH: methodology. EH: software, formal analysis, writing—original draft preparation, and visualization. EH and RR: validation, resources, supervision, and project administration. NE, GD, SS, SJ, QS, BM, EH, and RR: investigation. GD, SJ, SS, BM, and EH: data curation. RR and NE: writing—review and editing. All authors contributed to the article and approved the submitted version.

## Conflict of Interest

The authors declare that the research was conducted in the absence of any commercial or financial relationships that could be construed as a potential conflict of interest.

## Publisher's Note

All claims expressed in this article are solely those of the authors and do not necessarily represent those of their affiliated organizations, or those of the publisher, the editors and the reviewers. Any product that may be evaluated in this article, or claim that may be made by its manufacturer, is not guaranteed or endorsed by the publisher.

## Abbreviations

C: Mass concentration (mg/mL) of a constituent
ENDS: Electronic Nicotine Delivery System
F: Ratio of mass of a constituent to the mass of total particulate matter
HPHC: Hazardous or Potentially Hazardous Compound
MinAF: Minimum Aerosolization Flowrate
MaxAF: Maximum Aerosolization Flowrate
MinAD: Minimum Aerosolization Duration
MaxAD: Maximum Aerosolization Duration
PCU: Power Control Unit
PMTA, Premarket tobacco product application: U.S. Food and Drug Administration
TPM: Total Particulate Matter
Y: Mass yield (mg per puff) of a constituent.
